# Case Study: A Practical Application of an Aerosol Treatment in a Commercial Mill

**DOI:** 10.3390/insects10050150

**Published:** 2019-05-25

**Authors:** Deanna S. Scheff, Daniel Brabec, James F. Campbell, Frank H. Arthur

**Affiliations:** USDA—Agricultural Research Service Center for Grain and Animal Health Research Center, 1515 College Ave, Manhattan, KS 66502, USA; daniel.brabec@ars.usda.gov (D.B.); james.campbell@ars.usda.gov (J.F.C.); Frank.Arthur@ars.usda.gov (F.H.A.)

**Keywords:** Aerosol insecticides, commercial mill, *Tribolium confusum*, efficacy, deposition

## Abstract

In recent years, there has been an increasing interest and need for alternatives to structural fumigations, and one alternative that has been used across the industry is aerosol insecticides. Previous tests inside a pilot-scale mill demonstrated that aerosol particle size, delivery method, and the spatial configuration of the mill all influenced effectiveness. However, there is no research conducted inside large commercial facilities. The objective of this research was to evaluate a pyrethrin-plus-methoprene aerosol application inside a commercial mill on adult *Tribolium confusum* Jacquelin duVal, confused flour beetle, directly exposed to the aerosol and residual effects on larvae. Additionally, five aerodynamic particle sizer spectrometers were placed in the facility and recorded instantaneous spray concentration and estimated aerosol deposition. Adult *T. confusum* exposed nearest to the aerosol application points had the highest percentage of affected adults (>60%). The aerosol also had vertical movement when released at the top of a three-story open room; instantaneous concentrations were recorded on the ground floor. The aerosol residual was highly effective after 6-weeks post aerosol exposure, as 80% of the bioassays did not have any adult emergence from exposed larvae. This research demonstrates a practical use of aerosol insecticides and their potential to be an effective alternative to structural fumigations.

## 1. Introduction

Methyl bromide has been used worldwide as a fumigant since the 1930s, but has also been identified as an ozone-depleting agent under the Montreal Protocol, and its use as a structural fumigant for mills, processing plants, and warehouses is being phased out worldwide [1]. As a result, there is an increased interest and need for alternatives to methyl bromide fumigations to manage stored product insects inside structures. Alternatives to methyl bromide include but are not limited to physical control methods, including heat or cold treatments; fumigant replacements such as phosphine or sulfuryl fluoride; contact insecticides such as cyfluthrin or deltamethrin; or aerosols insecticides such as pyrethrin with pyriproxyfen insect growth regulator (IGR) or pyrethrin with methoprene-IGR. 

Aerosol insecticides are a suspension of a liquid chemical compound held under pressure, and when the pressure is relieved the released particles become atomized and disperse throughout a space before settling onto surfaces. Insecticides applied as aerosols are dispensed into the air as particles that commonly range from 5–50 µm [2]. The particle size distribution produced, distance traveled, and suspension time are all influenced by the chemical composition of the aerosol, the application or delivery method, and the ambient spatial configurations [3,4,5]. Aerosols are released from either a fixed dispensing system from overhead locations or from a portable cylinder or fogger and applied by certified pesticide applicators. Despite the different application methods, aerosol particles will not penetrate machinery, storage containers, packaging materials, or large accumulations of food particles. 

Aerosol particle size is a critical factor affecting the efficacy of insecticides, since smaller droplets tend not to settle onto surfaces or impinge on the insect cuticle. For example, laboratory testing in a continuous flow chamber has demonstrated how larger particle sizes of 16 µm were more effective than smaller particle sizes of 2 µm at preventing *Tribolium confusum* Jacquelin du Val, confused flour beetle, and *Trogoderma variabile* Ballion, warehouse beetle, development from the larval to the adult stage [6]. Particle size also impacts movement of particles within a structure, with larger droplets traveling shorter distances than smaller droplets. A study in a pilot-scale flour mill that measured particle size as a function of distance from release point demonstrated the average geometric mean diameters decreased with distance from the application point, with particle sizes ranging from 3.2 to 7.1 µm [4]. These studies used pyrethrin combined with an insect growth regulator pyriproxyfen (NyGard, TurboCide Py 75 w/IGR) and dispensed from a pressurized cylinder (0.7% pyrethrin, 0.2% pyriproxyfen, 5% PBO, Chem-Tech, Des Moines, IA, USA). Unlike isolated laboratory testing where aerosol particle size distributions can be controlled, the practical application of aerosols inside large structures can result in large variation of particle size distributions among locations and will directly influence efficacy of the aerosol insecticide for controlling stored product insects.

Populations of stored product insects can be found throughout all areas of a mill and present several challenges for aerosols treatments to be highly effective. First, there is spatial variation in distribution and most of the population is in hidden locations. Second, there is spatial variation in deposition of the aerosol particles on surfaces [3] and application locations can also influence pattern of aerosol distribution [5]. Efficacy is also impacted by the insecticide type and the target insect species and stage. The efficacy of aerosols such as pyrethrins and pyrethroids is primarily through insects coming into direct contact with flying or walking individuals, typically adults, while insecticides with good residual efficacy such as IGRs can have efficacy after deposition of particles on a surface so that larvae will subsequently encounter the aerosol residues. Given that most of the population is hidden during a treatment, aerosols most likely contribute to population reduction through residual persistence of the IGR component that is combined with pyrethrin. Exposures of late state larvae of *Lasioderma serricorne* F., the cigarette beetle on concrete arenas treated with a pyrethrin and methoprene formulation, resulted in no adult emergence of those exposed larvae, even after 8 weeks post-exposure [6]. 

Data from studies involving laboratory and research experiments in single rooms of small-scale mills have given researchers and pest control operators valuable insight on the effectiveness of aerosols as an alternative structural treatment option. However, there has been no published research conducted using aerosols inside a large multi-floor and multi-room commercial milling facility. The objective of this project was to evaluate a pyrethrin + pyriproxyfen aerosol application inside a multi-room and multi-floor commercial rice mill. We used adult *T. confusum* to determine the initial impacts of exposure, measured as number of affected adults and final mortality, and *T. confusum* larvae to determine the residual efficacy of the aerosol treatment. We also placedinstruments that measure particle size throughout the facility to monitor particle mass concentrations and predicted deposition. This experiment is designed to be used as an aid in understanding how the industry actively uses aerosols and the spatial variation in efficacy, as measured using particle concentration and deposition, and impact of direct treatment on susceptible *T. confusum* adults and residual activity of the IGR against susceptible *T. confusum* larvae.

## 2. Materials and Methods

### 2.1. Research Location 

The aerosol application for this study took place in a commercial rice milling facility during the summer of 2016. The ambient temperature outside on the day of testing was approximately 29–33 °C. The specific name, location, and specific details about the rice mill cannot be disclosed due to the confidentiality agreement required to conduct research on the property. Due to the complexity of the facility, it was estimated that the volume of space treated to be greater than 25,000 m^3^, and a generalized description of the rice milling facility is as follows. 

**Figure 1:**
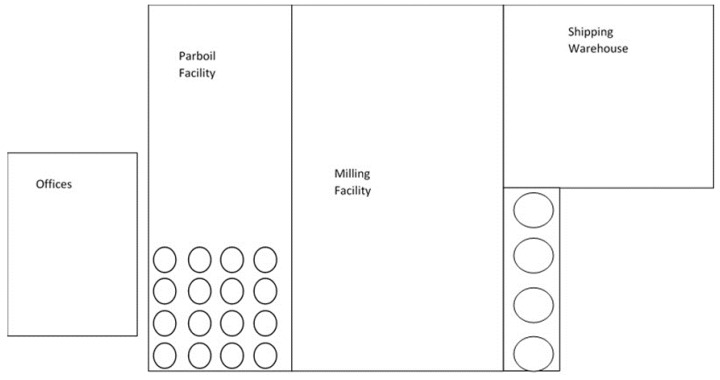
General layout of milling complex tested.

The milling complex consisted of three interconnected buildings that were treated: the parboil facility, milling facility, and shipping/warehouse facility (Figure 1). The first building on the east side of the complex was the parboil facility, which was three stories tall with half of the space containing several large and tall rice storage bins, while the other half had parboiling equipment located on the first flour. The parboil facility was considered an open concept layout, with only grated walkways, flooring, and stairs to allow access to the top of the storage bins and movement up and down and throughout the air space. 

The second building of the complex was the main milling facility, containing three separate and distinct floors and rice processing equipment, storage bins, and product packaging equipment. The third floor was laid out in a rectangular shape. On the south side of the third floor was a completely enclosed room that contained the old rice flour milling equipment, while on the northwest side of the third floor there was a doorway to the tops of four large rice storage bins. The room was termed large bin room. This storage area contained four large bin storage units. There was only one entry point, through a doorway, and the rest of the bin room was closed off from the third floor. There was a small walkway access to the top of the bins, but the remainder of the space was fully open to the first floor below, which was over 20 m below. The remainder of the third floor contained conveyers, structural beams, milling equipment, and the facility control room. 

The second floor of the milling facility contained rows of rice milling equipment. On the west side of the second floor there was the finished rice packaging room. This area contained the filling and bagging equipment, conveyers, and other packaging equipment used to create a finished product that would be ready to ship. The bagging room was separated from the second floor by large, vertical plastic curtains. The first floor of the milling facility contained additional conveyer systems, structural beams, milling equipment, and machinery. On the northwest side was an access doorway to the bottom of the large bin storage, described previously. On the southwest side was access doorway to the shipping and receiving warehouse, which was separated by vertical plastic curtains. 

The third building was the shipping and receiving warehouse. The warehouse was three-four stories high, with rows of storage racks that held large wooden pallets of finished products. The warehouse also contained two loading docks, on the first floor, where products could be loaded or unloaded from semi-trailers. The milling complex also had one building with administrative offices located next to the parboil facility. This building was separate from the milling complex and was not treated.

### 2.2. Bioassays and Particle Size Measurements

The *T. confusum* used in the study were from a pesticide-susceptible strain that has been maintained at the United States Department of Agriculture–Agricultural Research Service–Center for Grain and Animal Health Research (USDA-ARS-CGAHR) in Manhattan, KS, USA, for over 30 years. The *T. confusum* colony was reared on a diet of 95% whole wheat flour plus 5% brewer’s yeast and maintained in an environmental chamber at 27 °C and 60% r.h. in complete darkness. 

**Figure 2:**
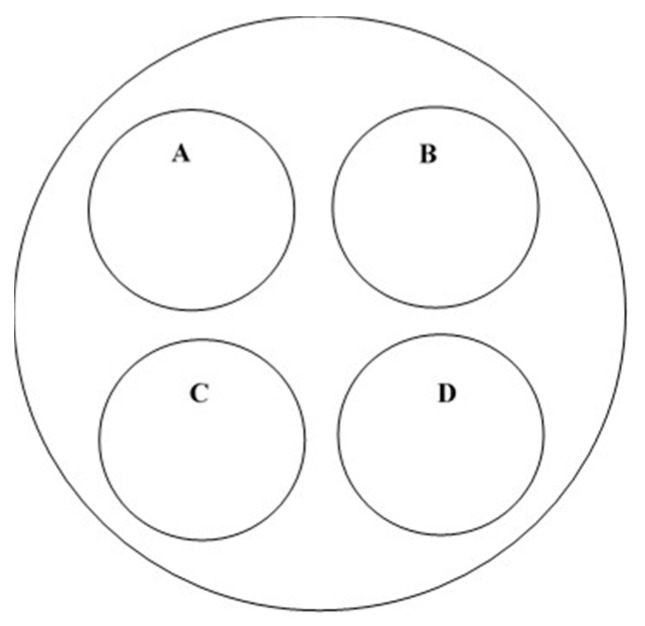
A 150 × 25 mm plastic Petri dish containing four concrete bioassay arenas: (**A**) bioassay arena containing 0.40 ± 0.01 g of *T. confusum* diet plus 10 adult beetles; (**B**) bioassay arena used for two week residual testing; (**C**) bioassay arena used for four week residual testing; and (**D**) bioassay arena used for six week residual testing.

Bioassay arenas were prepared as described in previous research [7]. Briefly, a driveway patching material (Rockite, Hartline Products Co., Inc., Cleveland, OH, USA) was mixed with water to create a slurry. The slurry was poured in to the bottom 0.5 cm of a 60 × 15 mm plastic Petri dish (~ 22cm^2^) to create concrete bioassay arenas. The arenas were allowed to dry at ambient conditions for a minimum of 48 h before use. Four individual treatment arenas were placed inside a larger 150 × 25 mm plastic Petri dish (~170 cm^2^) as described in Figure 2, to create one larger bioassay arena, and the group of arenas were placed at one of 20 locations throughout the rice-milling complex. An additional 10 bioassay arenas were prepared in the same manner and were used as untreated controls. Five bioassay arenas were placed in the office building, which is adjacent but separated from the parboil facility. Five additional bioassay arenas were placed in the milling control room located on the second floor of the milling facility. This area was closed off during application and no aerosol was applied due to computerized equipment. During aerosol applications, both entry/exit doors were shut to prevent aerosol particles from entering the room. 

At each location, one of the four individual arenas (Figure 2A) was used to determine the initial effect of the aerosol on adult *T. confusum*; the three remaining arenas were used to determine the residual effect of the aerosol on 4-week old *T. confusum* larvae at two, four, and six weeks post aerosol treatment. Prior to the aerosol treatment, 0.40 ± 0.01 g of *T. confusum* diet was added to the testing arena along with 10 mixed-sex, 1–2-week-old adult *T. confusum*. The three remaining concrete arenas did not contain diet. Following the aerosol application, the number of adult beetles that were affected in each arena was recorded and converted to a percentage (out of 10). Affected adult beetles were defined as on their backs, being unable to right themselves, or having impaired or no movement when observed. 

After the aerosol application, the bioassay dishes were placed in large plastic tubs and transported back to the USDA-ARS-CGAHR facilities in Manhattan, Kansas, USA, where they were held in an environmental chamber set at 27 °C and 60% r.h. in complete darkness. Bioassay dishes containing adults were observed after 7 d post aerosol treatment, and the beetles were classified as live, affected, or dead, and converted to a percentage (out of 10). 

The remaining concrete dishes exposed during the aerosol applications (Figure 2B–D) were used to conduct two, four, and six-week residual testing, respectively, of the aerosol insecticide against 4-week old *T. confusum* larvae. At these times, ten 4-week old larvae were placed on a concrete arena along with 0.40 ± 0.01 g of diet and monitored for adult emergence at 27 °C and 60% r.h. for up to 4 weeks. After 4 weeks, bioassay dishes were examined, and life stages were categorized as larvae, pupae (dead and/or deformed), deformed adults (twisted wings, split wings, missing elytra, etc.), or normal adults.

The aerosol was also evaluated by measuring the distribution of different aerosol particles (droplets) distributed to five different locations, using Aerodynamic Particle Sizer (APS) spectrometers (TSI Incorporated, Shoreview, MN, USA). Each APS location also had a corresponding bioassay arena. Each APS unit samples the air and counts the number of particles of different sizes over 20 second intervals. Data reported is the counts over an 80-minute period, ~240 sampling events, since after this period only small particles that did not settle remained in the air and there were no significant changes in particle sizes recorded. The count data were converted to mass concentration (mg/m^3^) per 20-second interval. Deposition was estimated from the combination of mass concentration and estimated settling of aerosol particles for each 20 second event. The total deposition per instrument location was the summation over the 80-minute sampling period. 

### 2.3. Aerosol Treatment Positions and Bioassay Arena Locations

The aerosol insecticide was applied from different release points, as described below, throughout the entire milling complex, which includes the parboil room, three separate milling floors, packaging room, large bin room, and the shipping and receiving warehouse. The aerosol used in the study was a cylinderized pyrethrin (0.7%), piperonyl butoxide (5.0%), pyriproxyfen (0.267%), and other ingredients (94.033%), with a carbon dioxide (CO_2_) carrier (TurboCide Py-75 with IGR, ChemTech Ltd., Des Moines, IA, USA).

The aerosol application was done by a certified pesticide application. All doors and windows were closed and sealed, and the air ventilation system was shut off, in accordance to company policy. The aerosol application started at 08:00 hours, and the final aerosol release was at ~09:00 hours. The treatment holding period was approximately 2 hours, followed by a ventilation period to aerate the facility and remove any remaining aerosol particles. After the pesticide applicator deemed the facility to be safe, approximately 2 hours after final aerosol release, all bioassay dishes were collected. The entire aerosol application time from the start of application until all bioassay dishes were collected was 5 hours.

The cylinder containing the aerosol used for this field study was placed on a wheeled cart and released at 10 different locations throughout the complex, starting at the third-floor locations and moving downward to the first floor. The application location descriptions, along with bioassay locations, are described as follows. 

Aerosol application location 1 was on the third floor in the old flour room and had the corresponding bioassay arena 1. Application location 2 was in the large bin room, with bioassay arenas 2, 3, and 4. Bioassay arena 2 was on the top of a bin on the third floor, while arena 3 was directly below arena 2 on the first floor and arena 4 was on the first floor but inside the bottom portion of one of the storage bins. These locations were used to determine if the aerosol was able to disperse downward to the first floor, when only released from the third floor. It was also used to tested if the aerosol was able to travel underneath/around the bin structures themselves. 

Application location 3 was on the third floor of the milling building. The applicator released the aerosol in the middle of the floor and directed the spray to the east and west. Bioassay arenas 5, 6, and 7 were located on this floor. Arena 5 was located in the middle of the third floor along with APS unit #1, and along the route of the spray application. Arena 6 was located to the east of the aerosol release position, and arena 7 was on the western side of the floor between two beam structures. Arenas 5, 6, and 7 were all roughly in a linear line with each other, with arena 7 approximately twice the distance from the aerosol release position compared to arena 6.

Application location 4 was in the parboil room on the third floor, where bioassay arenas 8, 9, and 10 were located. Arena 8 was located on the top of the large bins, at roughly a 45° angle from the aerosol release point along with APS unit #2. Arenas 9 and 10 were located on the first floor of the parboil room. Arena 8 was located directly in front of where the aerosol was released from. This arena was designed to determine how effectively the aerosol dispersed downward through the metal grating. Arena 10 was also located on the first floor of the parboil room, underneath beam structures, along with APS unit #3.

Application location 5 was in the packaging room, along with bioassay arenas 11 and 12. Arena 11 was located in the middle of the floor, in a completely open position. Arena 12 was at a 90° angle from arena 11 and located underneath the conveyor system used for the packaging process. Application location 6 was on the 2nd floor in an arena deemed a "hot spot" by the milling manager. This location contained a large duct that went to the outside, and insects were previously spotted on the outside structure at this location. Bioassay arena 13 was placed directly underneath the duct work. Application location 7 was on the second floor and was associated with bioassay arenas 14 and 15. Both bioassay arenas were placed in-between the two rows of milling equipment. Arena 14 was on the western side of the floor, along with APS unit #4. Arena 15 was on the eastern side of the floor.

Application location 8 was in the warehouse building, specifically near the loading docks, and bioassay arena 16 was located in front of the loading docks. Application location 9 was also in the warehouse building, specifically in the section that held the rows of pallet racking. Bioassay arenas 17, 18, and 19 were located in this section. All bioassay arenas were placed in a straight line. Arena 17 was place in the middle of the floor. Arena 18 was placed on the outside edge of the racking system. Arena 19 was placed in the middle of the pallet racking system. Application 10 occurred on the first floor and was the final aerosol release position for the building. Arena 20 was located in an open position in the middle of the floor space, along with APS unit #5.

Exact distances between aerosol release positions and bioassay dishes could not be determined, because only the certified application could be in the building at the time of application. The certified applicator gave approximate positions of where they would release the aerosol, but the positions could vary. In addition, exact amounts of aerosol released at each position could not be determined. The cylinderized aerosol was equipped with a valve that registered open or closed and did not contain any mechanical or electrical readouts. The amount of aerosol used in the test was determined from label rates and the total volume of the three building facility. The certified applicator would stop at each pre-determined position and release some insecticide and then move on towards the next location.

Only one aerosol treatment was measured in the facility, due to constraints in scheduling and distance, so this analysis should be considered a case study of an aerosol application conducted on a large commercial scale. The multiple sampling locations will enable us to evaluate the variability aerosol particles among different locations in a facility, but this analysis needs to be viewed in conjunction with laboratory, small scale, controlled experiments from previous research [3,5,7,8,9,10]. 

## 3. Results and Discussion

### 3.1. Effect of Aerosol Dispersal on T. confusum Adults

**Figure 3:**
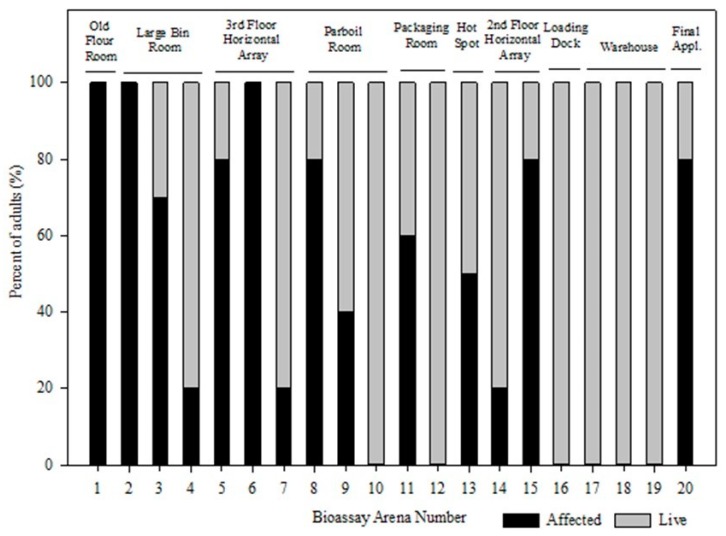
The percent of live vs. affected *T. confusum* adults after initial exposure to aerosol applications at each bioassay arena location.

The effect of the aerosol application varied among different areas within the rice mill as well as the with the sequence of application locations (first location vs. middle locations vs. last location), as depicted in Figure 3. The insecticide treatment started on the third floor of the processing building, old rice flour mill, and resulted in 100% affected adults (bioassay 1), whereas the applications near the end, locations 16–19, resulted in no affected adults. As the aerosol applications progressed from the first application to the last application, the number of affected *T. confusum* adults was reduced. This could indicate the amount of insecticide released differed between the first and last application point, either because the applicator opened the valve and released more product at the different locations, or that the tank was getting low and although the release time was sufficient the amount of active ingredient left in the cylinder was insufficient to cause efficacy. Bioassay arena 20 had 80% affected adult *T. confusum*, even though it was at the end of the aerosol treatment. This bioassay location would have been close to the final release location, at which point the pesticide applicator released all the remaining ingredients in the cylinder. 

**Figure 4:**
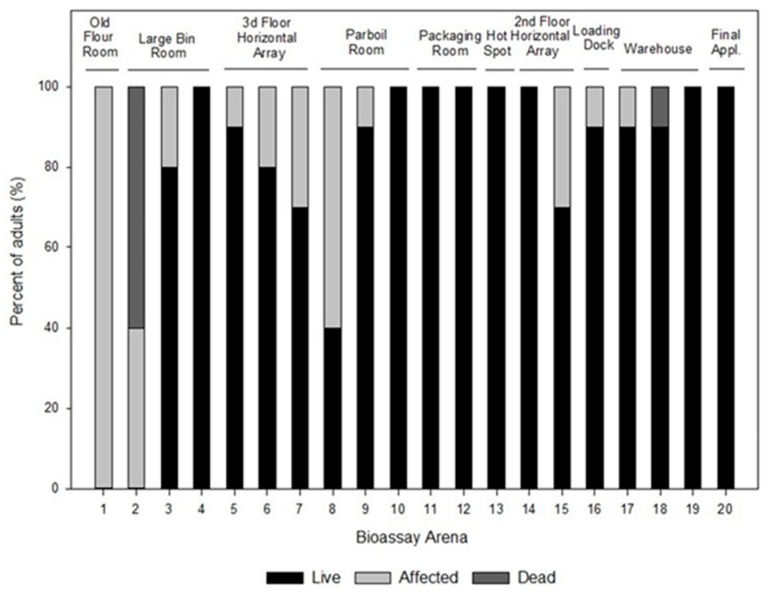
The percent of live vs. affected vs. dead adult *T. confusum* one week post aerosol application at each bioassay arena locations.

After one-week post aerosol application, adults in all the bioassay arenas were observed and number of live vs. affected vs. dead individuals was determined. Bioassay arena 1, where the first aerosol release occurred, was the only location that had 100% affected adults after one week, and among all other bioassay arenas the percentage of affected adults ranged from 0–60% (Figure 4). Only two locations had dead adult *T. confusum,* and at many locations the initially affected adults recovered and were mobile after this one-week recovery period. Eight out of the 20 locations had complete adult recovery after a week. The ability of affected adult *T. confusum* to recover as observed in this study is consistent with previous research [5,6,8,10]. The affected state, or knockdown as it is sometimes termed, is a transitional category from which individual insects may survive or die, with the outcome likely related to the dosage of insecticide received [8]. In order for the pyrethrin component of the aerosol to be effective on adult *T. confusum,* it needs to penetrate the insect’s cuticle in a high enough concentration to affect the nervous system and permanently bind to sodium channels and disrupt their function [11]. The efficacy of initial aerosol applications relies heavily on adult stored product insects coming into direct contact with the aerosol particles by walking, flying, or ingesting food particles contaminated with the insecticide. This ability of *T. confusum* adults to recover from the affected state further emphasizes that once an aerosol is applied, further integrated pest management (IPM) practices still need to occur. This includes, sanitation, trapping, and monitoring the insect population levels after a treatment is applied. 

**Figure 5:**
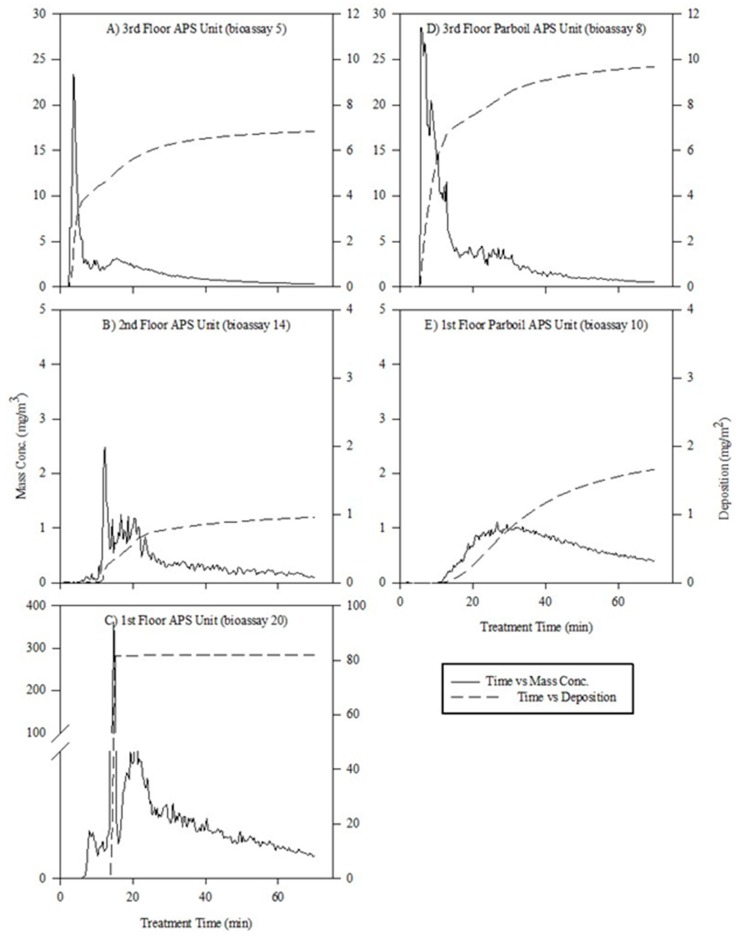
A comparison of the mass concentration (mg/m^3^, left y-axis, solid line) and estimated particle deposition (mg/m^2^, right y-axis, dashed line) during the aerosol treatment time (x-axis). Data is from (**A**) Aerodynamic Particle Sizer Spectrometers (APS) unit located on the third floor of the milling facility; (**B**) APS unit located on the second floor of the milling facility; (**C**) APS unit located on first floor of the milling facility; (**D**) APS unit located on the third floor of the parboil facility; and (**E**) APS unit located on the first floor of the parboil facility.

From the data analyzed from the five different APS units located throughout the entire facility, it was apparent that during the initial aerosol application there was a high concentration of particles in several locations; however, the concentration of the particles quickly diminished as the applications proceeded (Figure 5A–E). Only two locations had relatively high deposition (Figure 5A,D), but total deposition was much lower compared to results from a previous study where aerosol particle deposition was reported at ~58 mg/m^2^ [4] using the same aerosol formulations and the APS unit was placed 4.3 m away from the application site.

Data on mass concentration showed differences between application points as well, as there was a trend for higher particle concentration and deposition at the third-floor application point (APS unit #1) (Figure 5A) than on the second-floor location (APS unit #4) (Figure 5B). APS unit #1 was located near the applicators path and received significantly more insecticide particles, whereas the APS unit #4 was located between rows of milling equipment so path of aerosol particles was more likely to be obstructed. Based on the bioassay data on adult mortality, it was predicted the first-floor location would have a lower concentration and deposition. The location of the APS unit was the only location on the first floor with affected adults, and there was also a large spike in the particle concentration data (Figure 5C). This corresponds to the high percentage of affected adults at the corresponding bioassay 20 (80%). Results indicated that some chemical was received at this location, but it was not a fine particle plume as in the earlier locations (Figure 5C); rather, it was composed of larger and fewer particles of insecticide that did not travel as far. If the tank was nearly empty or contained little insecticide at this location, this could have generated this pattern of particle distribution and the high percentage of affected adults compared to all other locations on the first floor of the facility. However, all adult *T. confusum* recovered seven days post application, indicating that the large plume of particles that was recorded on APS Unit #5 was not sufficient to kill the exposed beetles. 

Previous research [3,4,5] has shown that efficacy of aerosols declines with horizontal distance from the aerosol release point. As this was a multi-floor building with an open structure, we were able to show how the effect of the aerosol application varied vertically within the mill as well. Generally, when aerosols were released at the top of a multi-story space particle, mass concentration and deposition and percentage of affected adults decreased with distance downward. Looking specifically at bioassays 2, 3, and 4, the effect on adult beetles decreased as the height differential from the aerosol application increased (or the distance the aerosol particles had to traverse increased). The distance from bioassay 2 (top) to bioassay 3 (bottom) was approximately 15 m, and the percentage of affected adults decreased from 100% to 75% in open areas and decreased to 20% in slightly obstructed areas (bioassay 4). Similar phenomena were observed in the parboil room, bioassays 8–11, where the aerosol was only applied on the third floor of the facility and was expected to drift down to the ground level. Bioassay 8 had 80% affected adults, whereas bioassay 9 and 10 had 40 and 0%, respectively. The high percentage of affected adults on bioassay arena 8 correlates to a high mass concentration of aerosol particles recorded by the APS unit #2 (Figure 5D). The high concentration of particles indicates there was a large amount of insecticide present in the air space that could potentially deposit on the body of the adult *T. confusum*. Conversely, the mass concentration and calculated deposition recorded with APS unit #3 on the first floor of the parboil room showed approximately a 90% reduction (Figure 5E) and could account for the lack of affected *T. confusum* adults. 

Once the aerosol is released into a space and deposited on surfaces, the degradation of the pyrethrin begins, and it will generally lose all efficacy within a matter of days. However, when a pyrethrin is paired with an IGR, as in this study, the IGR component persists on surfaces for longer and can give residual protection of the surfaces that have been treated [7]. In the current study, as well as previous studies, adult *T. confusum* were used as an indicator of the initial effectiveness and as a metric to determine variation in the distribution of the aerosol treatment. The use of *T. confusum* in this study helped to illustrate areas across the facility that may not have received adequate dosages of the aerosol insecticide. Increasing the number of application points, improving air movement, releasing more product, or being more aware of the level of insecticide in the cylinder could be employed by applicators to increase the effectiveness of the aerosol application. The aerosol insecticide used in this study also contained an IGR component; the ensuing residual bioassays using larvae of stored product insects give a more complete picture of the effectiveness of the treatment. 

### 3.2. Residual Effect of Aerosol Applications on T. confusum Larvae

**Figure 6:**
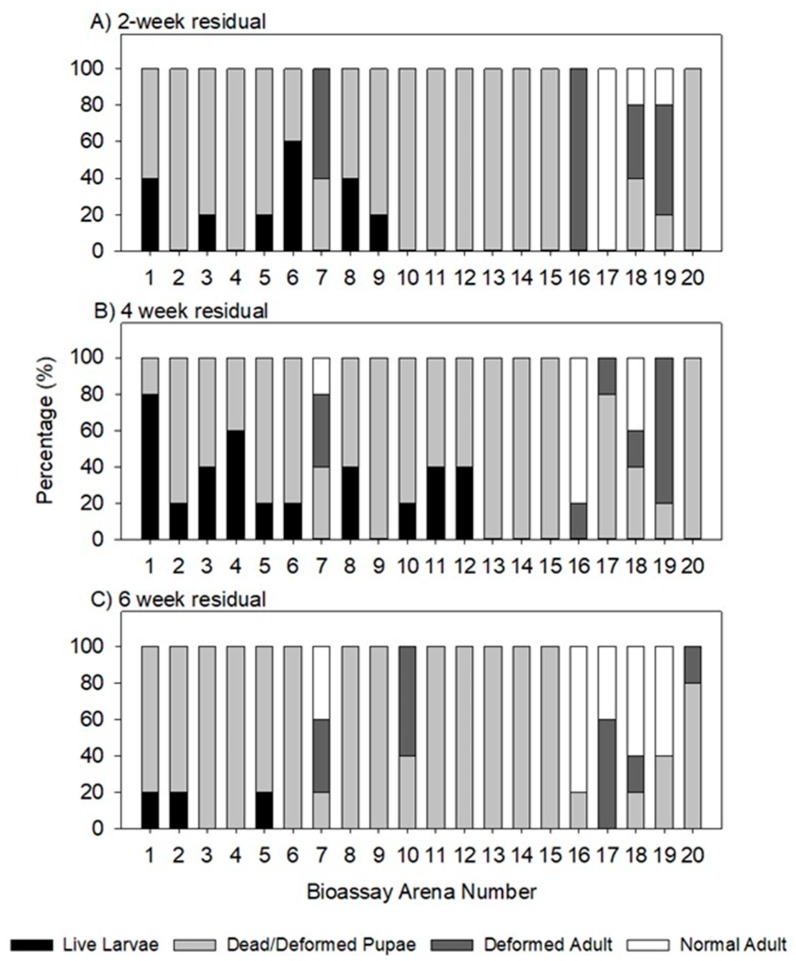
The percent of larvae, pupae (dead or deformed), deformed adult, or normal adults after larvae were exposed to aerosol exposed concrete arenas (**A**) two weeks post aerosol treatment; (**B**) four weeks post aerosol treatment; and (**C**) six weeks post aerosol treatment.

The percentage of adults emerging from larvae exposed in untreated control arenas at two, four, and six weeks post aerosol treatment ranged from 86–96%. Comparatively, in the aerosol treated areas after two weeks post aerosol treatment there were only three locations, bioassays 17–19, in which normal adult *T. confusum* emerged (Figure 6A). Bioassay arena 17, located in the shipping room, had 100% normal adult emergence, but in the four and six-week post aerosol treatments (Figure 6B,C) there was less adult emergence. This suggests some unevenness in particle deposition across the concrete bioassay arenas at the same location. Among all bioassay locations, those located in the loading dock and warehouse area of the facility (bioassays 16–19) appeared not to have received a significant amount of aerosol particle deposition, which resulted in adult emergence for all residual time points tested. As discussed before, these locations were the last ones treated and the cylinder may have no longer been releasing sufficient active ingredient In contrast, those bioassay areas near the beginning of the aerosol treatment were strongly affected by the aerosol treatment and resulted in 100% suppression of normal adult emergence at two weeks post treatment. Additionally, only bioassay arena 7 had any normal adult emergence after four and six-weeks post treatment and ranged from 20–40%, respectively. 

There was greater variation in aerosol treatment efficacy, and generally lower efficacy, when evaluating impact on adult *T. confusum* after one week post exposure, in comparison to evaluation of the IGR residual efficacy when measured using *T. confusum* larvae up to six weeks post treatment. Similar results were obtained for *T. confusum* and *T. castaneum* (Herbst), the red flour beetle, where adult emergence of larvae exposed on treated concrete arenas was significantly lower compared to untreated arenas for up to seven weeks post treatment [7]. Where the aerosol was applied at the top of an ~15 m space, the particles dispersed downward and were deposited on the surface of the concrete arenas on the bottom floor and inhibited adult emergence for up to six weeks. This was evident by comparing bioassay locations 2–4 and 8–10 (Figure 6A–C), whereby the bioassays on the top floor where the aerosol was applied (arenas 2 and 8) resulted in 100% suppression as well as those arenas on the bottom floor where the aerosol had to settle (arenas 3, 4, 9, and 10). 

While there is likely to have been variation in the amount of IGR deposited on surfaces, as indicated by the APS data, the range of active ingredient deposited appears to be in most cases well above that needed to cause complete disruption of larval development. A particle deposition of approximately 2 mg/m^2^ was enough to inhibit larval development on bioassay arena 10 for up to 4 weeks post treatment (Figure 5E). As the length of aerosol treatment increased in this field study, the amount of particle deposition increased as the aerosol particles began to settle on the flooring surface. This result corresponds to previous research using APS units in laboratory tests [9] and in a pilot-scale mill [4]. The deposition values calculated in the current study are lower than the research conducted in the pilot-scale mill [4], whereby the highest deposition in the current study was roughly 10 mg/m^2^ and in the previous study was roughly 125 mg/m^2^. This indicates that the effective rate applied was lower in the commercial location than in the field tests, likely due to the much larger volume being treated. Despite the lower particle deposition and mass concentration, the aerosol insecticide treatment had an effective residual impact on development of susceptible *T. confusum* larvae. The use of an IGR component in the aerosol formulation gave good residual efficacy in many areas of the facility. The positive implications of using an IGR is the residual insecticide are specific to insects. IGRs act specifically on the juvenal hormone in insect species and disrupt their life-cycle, and are practically nontoxic to humans. According to the pyriproxyfen label, it can be applied as a space spray, for treatment of stored food areas, airspace above dried fruit products, and stored food products.

Despite the residual control of *T. confusum* larvae throughout the facility, if greater and more consistent direct mortality from the pyrethroid is desired it may be necessary to apply more material, perhaps using multiple cylinders to treat the space. Additional considerations that aerosol applicators should consider are using a gauge to directly measure the amount of insecticide being applied in a give space, increasing the number of application points, or using some-type of fan or air circulation system to increase movement of the aerosol particles. The results of this study demonstrate that many assumptions when applying aerosols are not true, such as the amount of horizontal and vertical movement of aerosol particles as indicated by the treatment in the parboil facility. Understanding aspects such as this will aid applicators in re-thinking how applications are made and changes that could be implemented to increase efficacy of treatments. This experiment should be used as an aid to industry and researchers on how aerosols are being used in the field and were improvements and further research are needed.

## 4. Conclusions

Aerosol insecticides have a number of attributes, including relatively low cost, minimal shutdown time, ease of use, portability, and residual effectiveness, but our results show that spatial variation in effectiveness can occur and that strategies to improve the consistency of coverage could improve effectiveness. Susceptible adult and larval *T. confusum* are useful tools to demonstrate this variation in coverage; however, because the IGR was effective at very low dosages, the practical impacts of this variation in deposition are in part mitigated. Pairing pyrethroid and pyrethrin insecticides with an IGR is a common practice, and while aerosol treatments with pyrethroid/pyrethrin alone do occur, they it is much less frequent for IGRs to be applied alone. This study also illustrates how application strategies can impact the effectiveness of the treatment and highlights some of the limitations in aerosol particle movement that would be useful for practitioners. Further studies conducted in large-scale mills, warehouses, and/or storage areas are needed to enhance the research information available for further improvements for pest management.
